# IFN-*γ* induces aberrant CD49b^+^ NK cell recruitment through regulating CX3CL1: a novel mechanism by which IFN-*γ* provokes pregnancy failure

**DOI:** 10.1038/cddis.2014.470

**Published:** 2014-11-06

**Authors:** Z-Y Li, H-H Chao, H-Y Liu, Z-H Song, L-L Li, Y-J Zhang, Y Yang, J-P Peng

**Affiliations:** 1 State Key Laboratory of Reproductive Biology, Institute of Zoology, Chinese Academy of Sciences, Beijing, People's Republic of China; 2 University of Chinese Academy of Sciences, Beijing, People's Republic of China

## Abstract

Interferon-*γ* (IFN-*γ*), a pleiotropic lymphokine, has important regulatory effects on many cell types. Although IFN-*γ* is essential for the initiation of uterine vascular modifications and maintenance of decidual integrity, IFN-*γ* administration can also cause pregnancy failure in many species. However, little is known about the effector mechanisms involved. In this study, using an IFN-*γ*-induced abortion mouse model, we reported that no *Dolichos biflorus agglutinin* lectin-positive uterine natural killer (uNK) cells were observed in the uteri from IFN-*γ*-induced abortion mice. By contrast, the percentage of CD3^−^CD49b^+^ NK cells in the uterus and blood from a foetal resorption group was significantly higher than that of the control group. Similarly, significantly upregulated expression of CD49b (a pan-NK cell marker), CX3CL1 and CX3CR1 (CX3CL1 receptor) was detected in the uteri of IFN-*γ*-induced abortion mice. Using isolated uterine stromal cells, we showed that upregulated expression of CX3CL1 by IFN-*γ* was dependent on a Janus family kinase 2-signal transducers and activators of transcription 1 (JAK2-STAT1) pathway. We further demonstrated the chemotactic activity of CX3CL1 in uterine stromal cell conditioned medium on primary splenic NK cells. Finally, we observed increased recruitment of CD49b^+^ NK cells into the endometrium after exogenous CX3CL1 administration. Collectively, our findings indicate that IFN-*γ* can significantly increase uterine CX3CL1 expression via activation of the JAK2-STAT1 pathway, thus inducing CD49b^+^ NK cell uterine homing, and eventually provoke foetal loss. Thus, we provide a new line of evidence correlating the deleterious effects of IFN-*γ* on pregnancy with the aberrant regulation of CX3CL1 and CD49b^+^ NK cells.

Interferon-*γ* (IFN-*γ*), as a proinflammatory cytokine produced in the uterus during early pregnancy, initiates endometrial vasculature remodelling and contributes to the normal health of the deciduas.^1^ However, IFN-*γ* administration can also cause pregnancy failure in rabbits^2^ and in mice.^3^ We previously demonstrated that the deleterious effects of IFN-*γ* were associated with the aberrant expression of major histocompatibility complex class II molecules^2, 4^ and increased apoptotic death of placental cytotrophoblast cells at the maternal–foetal interface.^5, 6^

Increasing evidence showed that natural killer (NK) cells had a critical role in foetal resorption, because the depletion of NK cells by anti-asialoGM1 Ab could reduce abortion rates.^7, 8, 9^ However, heavily uterine NK (uNK) cells were transiently found in the uteri of many species and could promote decidual transformation, vascularization and placental formation in midgestation.^10, 11^ In mice, CD49b (*α*2 integrin chain) is widely used as a pan-NK cell marker,^12^ whereas *Dolichos biflorus agglutinin* (DBA) lectin, which reacts with glycoconjugates containing *N*-acetylgalactosamine, is used as a specific uNK cell marker.^13^ With regard to the origin of uNK cells during pregnancy, Chantakru *et al.*^14^ demonstrated that the marked increase of uNK cells during decidualization was primarily caused by a remarkably increased recruitment of uNK cell precursors, but not *de novo* self-renewal of the existing uNK cells. However, the molecules attracting the homing of NK cells into the uterine bed during pregnancy remain unexplored.

Chemokines are a group of small, structurally related molecules that coordinate the homeostatic circulation of leucocytes.^15^ As an unusual member of the chemokine family, CX3CL1 consists of a chemokine domain attached to a glycosylated mucin-like transmembrane stalk^16^ and exhibits an efficient chemotactic activity for monocytes, T cells and NK cells.^17^ Interestingly, CX3CR1 (the CX3CL1 receptor) defines two killer lectin-like receptor G1-positive mouse NK cell subsets^18^ and regulates NK cell trafficking.^19^ In addition, IFN-*γ* could modulate the expression of CX3CL1 in endothelial cells.^20^ However, few data currently exist on the changes in CX3CL1 during pregnancy failure.

The purpose of the present study was to investigate whether IFN-*γ*-induced pregnancy failure was associated with the uterine production of CX3CL1 and NK cell uterine homing. We demonstrated herein that IFN-*γ* induced a highly increased proportion of CD49b^+^ NK cells in the uterus and peripheral blood and it induced a significantly upregulated uterine expression of CX3CL1. Additionally, our data showed that uterine CX3CL1 facilitated CD49b^+^ NK cell recruitment into the uterus. To our knowledge, this is the first evidence showing that IFN-*γ*, via stimulating uterine CX3CL1 production, induces CD49b^+^ NK cell uterine homing and thus pregnancy failure in mice.

## Results

### IFN-*γ* administration resulted in foetal resorption

**Figure 1:**
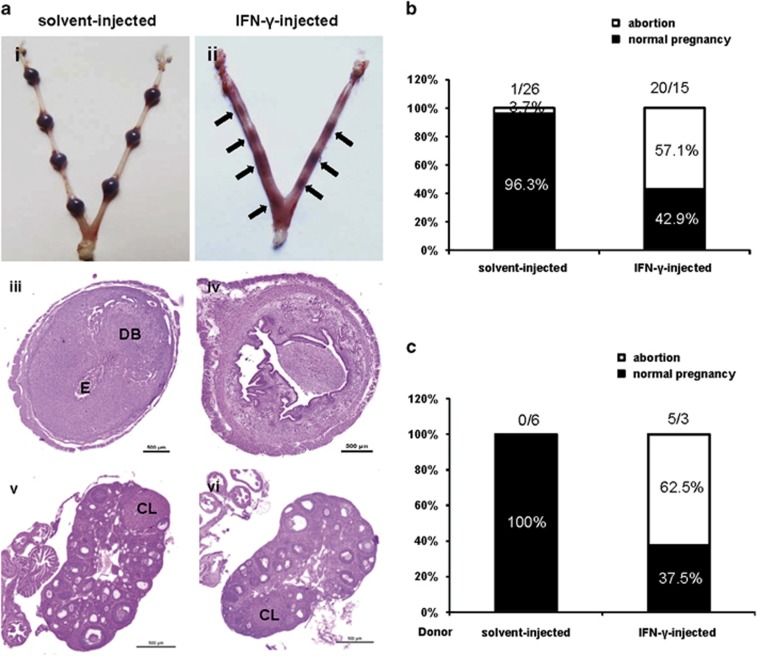
IFN-*γ* administration resulted in foetal resorption. Syngeneically mated BALB/c female mice were injected with solvent or IFN-*γ* intraperitoneally on GD6 and killed on GD8. (**a**) Representative macroscopic views of a healthy uterine horn from a solvent-injected mouse (i) and an aborted uterine horn from an IFN-*γ*-injected mouse (ii) are shown. H&E staining of uterine (iii, iv) and ovarian (v, vi) paraffin sections from solvent-injected mice and IFN-*γ*-injected mice are shown. Arrows indicate the corpus luteum. Photomicrographs are representative of at least three mice in each group. Scale bar: 500 *μ*m. (**b**) Ratio of foetal abortions induced by IFN-*γ* is shown. The numbers above the bars indicate the number of mice with abortion/normal pregnancy. The ratio of foetal abortions was calculated from the following formula: (no. of abortion/no. of abortion plus no. of normal pregnancy) × 100%. ***P<0.001 by *χ*2. (**c**) Splenic cells of placebo-treated or IFN-*γ*-induced abortion mice after erythrocyte lysis were transferred into syngeneically mated BALB/c mice on GD6, and mice were killed 2 days posttransfer. The ratio of foetal abortions is shown, and the numbers above the bars indicate the number of mice with abortion/normal pregnancy. The ratio of foetal abortions was calculated as above. CL, corpus luteum; DB, decidua basalis; E, embryo; no., number

To evaluate the adverse effects of IFN-*γ* on pregnancy, syngeneically mated BALB/c females received an injection of 5000 U IFN-*γ* intraperitoneally on gestational day 6 (GD6), and the incidence of foetal resorption was assessed 2 days after treatment. A dose of 5000 U IFN-*γ* was used after preliminary comparisons of different doses (data not shown). We observed that IFN-*γ* administration significantly increased the resorption rate (Figure 1b). The solvent control mice exhibited gross morphologically normal implantation sites (Figure 1ai). By contrast, IFN-*γ* at a dose of 5000 U resulted in embryo loss. Resorbing uterine contents were characterized by the degeneration of the decidua accompanied with thrombosis and haemorrhage. The remnants of decidual tissue had already passed into the uterine lumen with the embryos (Figure 1aii). Further histological examination of the control mice revealed a representative view of a GD8 embryo with well-developed deciduas and embryonic capsule (Figure 1aiii). By contrast, implantation sites from IFN-*γ*-treated mice displayed poorly developed deciduas and the absence of embryos (Figure 1aiv). However, the ovaries of IFN-*γ*-treated mice contained normal corpus luteum and exhibited no overt histological abnormalities compared with the control group (Figures 1av and vi). Interestingly, when the splenic cells of IFN-*γ*-induced abortion mice after erythrocyte lysis were transferred into syngeneically mated BALB/c on GD6, we also observed a 62.5% resorption rate 2 days posttransfer (Figure 1c), suggesting that IFN-*γ*-induced resorption was due to leucocytes. Thus, further experiments were designed to explore the causes underlying IFN-*γ*-induced resorption.

### IFN-*γ* treatment enhanced the accumulation of the CD49b^+^ NK cell subset

**Figure 2:**
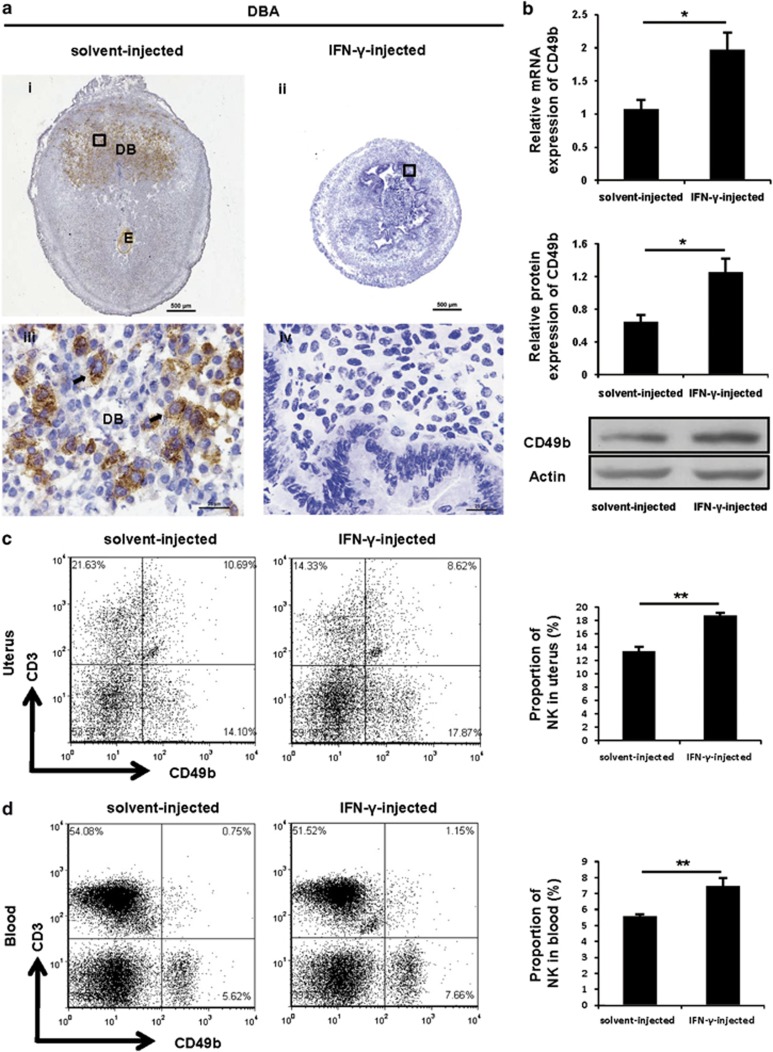
IFN-*γ* treatment enhanced the accumulation of the CD49b^+^ NK cell subset. Syngeneically mated BALB/c female mice were injected with solvent or IFN-*γ* intraperitoneally on GD6 and killed on GD8. (**a**) Analysis of DBA lectin-stained uNK cells in the uteri by immunohistochemistry. Arrows indicate DBA lectin-positive cells. Photomicrographs are representative of five mice in each group. Panels iii and iv are higher magnifications of areas marked by the black rectangles in panels i and ii, respectively. Scale bar: 500 *μ*m (i and ii) and 25 *μ*m (iii and iv). (**b**) CD49b expression *in uteri* was analysed by quantitative PCR (top panel) and western blotting (bottom panel). Data show the mean±S.E.M. of four independent experiments and are obtained from four mice of each group, respectively. *P<0.05 by independent samples *T*-test. Flow cytometric analysis of cell suspensions from uteri (**c**) and peripheral blood (**d**). See Supplementary Figures 1B and C for the gating strategy and the percentages of CD3^−^CD49b^+^ NK cells (lower-right quadrant). Data show the mean±S.E.M. of four (uteri) or five (blood) independent experiments and are obtained from four (uteri) or five (blood) mice of each group, respectively. **P<0.01 by independent samples *T*-test. DB, decidua basalis; E, embryo

Because uNK cells have critical functions in pregnancy,^21^ we examined whether IFN-*γ* treatment would alter the uNK cells. By performing immunostaining analysis, we found that the DBA lectin-positive cells were restricted to decidua basalis and mesometrial lymphoid aggregates of pregnancy (MLAp) of implantation sites in solvent control mice (Figures 2ai and iii). By contrast, no DBA-positive reaction was observed in the sections of uteri from IFN-*γ*-induced abortion mice (Figures 2aii and iv). Unexpectedly, CD49b was expressed at a significantly higher level in IFN-*γ*-treated mice (Figure 2b). Similar results were observed when CD49b expression was analysed on GD7 (Supplementary Figure 1A). Further, uteri were harvested to assess the percentage of CD3^−^CD49b^+^ NK cells in CD45^+^ leucocytes (see Supplementary Figure 1B for the gating strategy). The percentage of CD3^−^CD49b^+^ NK cells (lower-right quadrant) in the uterus from the IFN-*γ*-treated group was significantly higher than that from the control group (Figure 2c), as expected. Interestingly, a similarly significant increase was found in the peripheral blood (see Supplementary Figure 1C for the gating strategy) of IFN-*γ*-treated mice when compared with the control mice (Figure 2d). Overall, our findings suggested that IFN-*γ* induced a marked increase of CD49b^+^ NK cells in the uterus and peripheral blood.

### IFN-*γ* significantly increased uterine CX3CL1 expression via activation of the JAK2- STAT1 pathway

**Figure 3:**
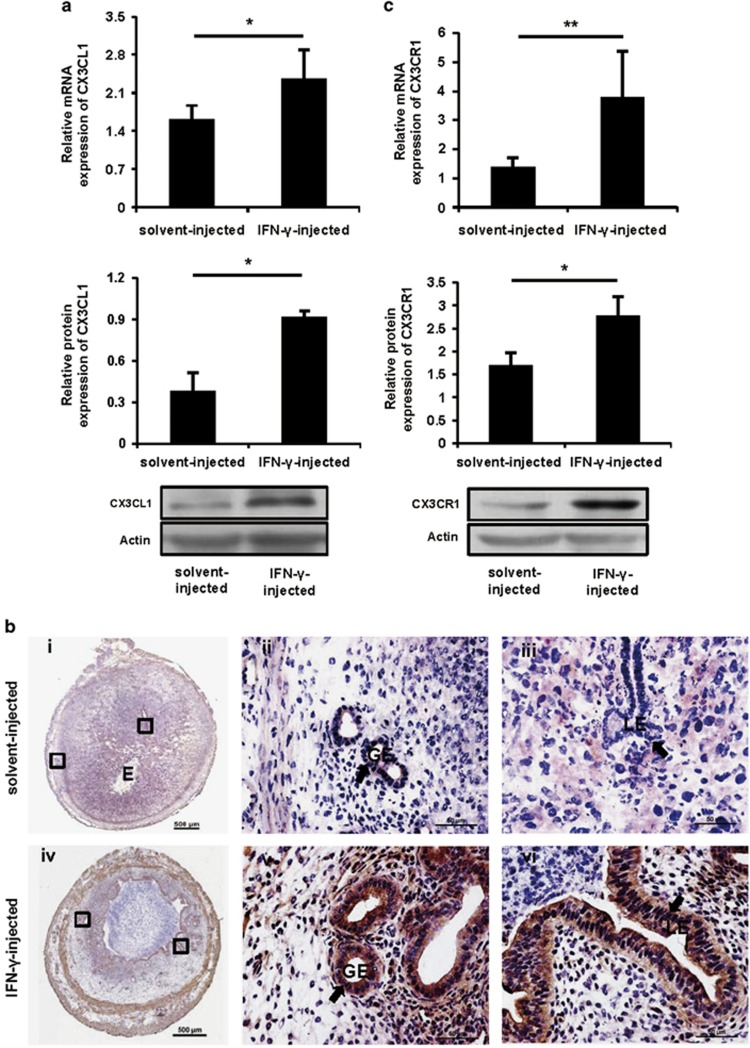
IFN-*γ* significantly increased uterine CX3CL1 and CX3CR1 expression. Syngeneically mated BALB/c female mice were injected with solvent or IFN-*γ* intraperitoneally on GD6 and killed on GD8. (**a**) CX3CL1 expression was analysed by quantitative PCR (top panel) and western blotting (bottom panel) *in uteri*. Data show the mean±S.E.M. of four independent experiments and are obtained from four mice of each group, respectively. *P<0.05 by independent samples *T*-test. (**b**) CX3CL1 expression was analysed by immunohistochemistry *in uteri*. Arrows indicate that stronger staining is observed in the LE and GE of the uteri from IFN-*γ*-injected mice. Photomicrographs are representative of three mice in each group. Panels ii, iii and v, vi are higher magnifications of different areas marked by the black rectangles in panels i and iv, respectively. Scale bar: 500 *μ*m (i and iv) and 50 *μ*m (ii, iii, v and vi). (**c**) CX3CR1 expression was analysed by quantitative PCR (top panel) and western blotting (bottom panel) *in uteri*. Data show the mean±S.E.M. of five (quantitative PCR) or three (western blotting) independent experiments and are obtained from five (quantitative PCR) or three (western blotting) mice of each group, respectively. *P<0.05 and **P<0.01 by independent samples *T*-test. E, embryo; GE, glandular epithelium; LE, luminal epithelium

To analyse whether the changes of NK cells were due to chemokines, quantitative PCR was performed to detect the expression of various chemokines in the uteri. The expression of CX3CL1 mRNA (Figure 3a, top panel) was markedly upregulated by IFN-*γ* treatment on GD8. The significantly enhanced expression of CX3CL1 was also confirmed by western blotting (Figure 3a, bottom panel). Furthermore, histological analysis revealed stronger staining in the luminal epithelium and glandular epithelium of the uterus from IFN-*γ*-treated mice compared with control mice (Figure 3b and Supplementary Figure 2A). Similarly, the expression of CX3CL1 protein was also upregulated in the uterus of IFN-*γ*-treated mice when analysed on GD7 (Supplementary Figure 2B). Interestingly, CX3CR1 expression was also significantly upregulated in the uterus from IFN-*γ*-treated mice (Figure 3c).

**Figure 4:**
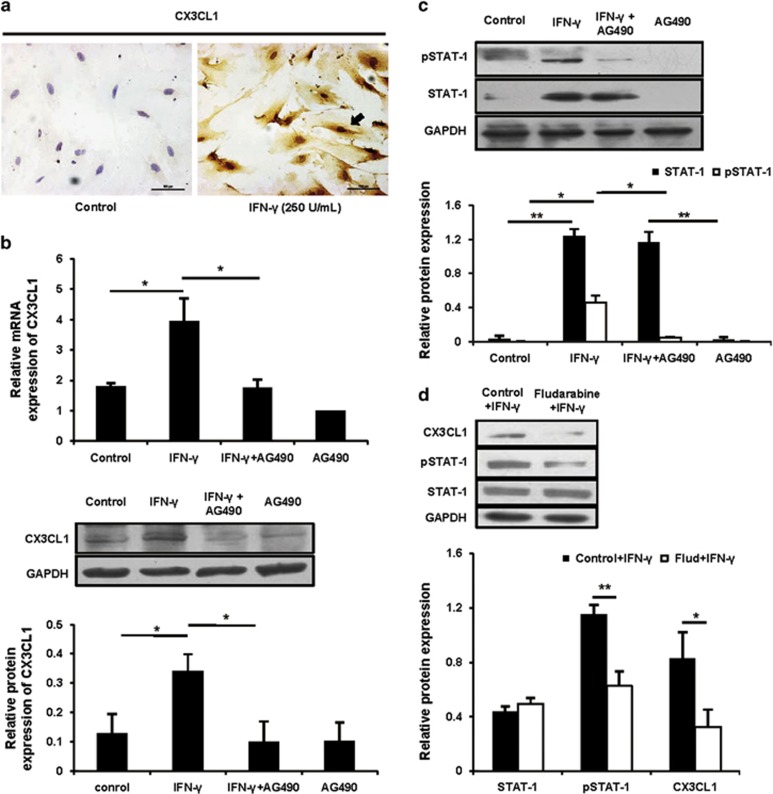
IFN-*γ* upregulated CX3CL1 via a JAK2-STAT1 pathway in uterine stromal cells. (**a**) The isolated uterine stromal cells were treated with or without IFN-*γ* at a dose of 250 U/ml for 12 h, and CX3CL1 protein expression was analysed by immunocytochemical staining. The arrow indicates that CX3CL1 protein expression is markedly induced in response to IFN-*γ*. Scale bar: 100 *μ*m. (**b**) Uterine stromal cells were pretreated with AG490 at 10 *μ*M for 2 h before IFN-*γ* treatment, and then CX3CL1 expression was analysed by quantitative PCR (top panel) and western blotting (bottom panel). Data show the mean±S.E.M. of three independent experiments, respectively. *P<0.05 by one-way analysis of variance (ANOVA). (**c**) The treatment was the same as described in (**b**). The STAT1 and pSTAT1 were analysed by western blotting and normalized to GAPDH and STAT1, respectively. Data show the mean±S.E.M. of three independent experiments. *P<0.05 and **P<0.01 by one-way ANOVA. (**d**) Uterine stromal cells were pretreated with fludarabine at 100 *μ*M for 2 h before IFN-*γ* treatment, and then CX3CL1, pSTAT1 and STAT1 were analysed by western blotting. CX3CL1, STAT1 and pSTAT1 were normalized to GAPDH, GAPDH and STAT1, respectively. Data show the mean±S.E.M. of three independent experiments. *P<0.05 and **P<0.01 by independent samples *T*-test

To further reveal how IFN-*γ* upregulated uterine expression of CX3CL1, we performed uterine stromal cell culture experiments. When uterine stromal cells were treated with IFN-*γ* at doses of 10, 100, 250 or 500 U/ml for 12 h, CX3CL1 protein expression was markedly induced in response to IFN-*γ* at a dose of 250 or 500 U/ml compared with the control group (Supplementary Figure 2C). Immunocytochemical staining displayed similar results (Figure 4a). When IFN-*γ* was administered at a dose of 250 U/ml, CX3CL1 expression levels varied in a time-dependent manner. The CX3CL1 expression level increased within 1 h, peaked at 6 h, continued for at least 12 h and then declined later (Supplementary Figure 2D). Thus, the treatment of IFN-*γ* at a dose of 250 U/ml for 12 h was applied in the following study.

Because the Janus family kinase-signal transducers and activators of transcription (JAK-STAT) pathway was widely investigated in IFN-*γ*-mediated signal transduction and transcriptional regulation signalling,^22^ we tested whether the regulation of CX3CL1 expression by IFN-*γ* occurred via the JAK-STAT pathway. When uterine stromal cells were pretreated with AG490, a specific JAK2 inhibitor,^23^ there was a dose-dependent inhibition of CX3CL1 upregulation by IFN-*γ* (data not shown). At 10 *μ*M, AG490 completely abrogated IFN-*γ*-mediated responses in uterine stromal cells (Figure 4b), suggesting that JAK2 mediated the IFN-*γ*-stimulated CX3CL1 expression. However, incubation with AG490 alone had no effect on the CX3CL1 basal level (Figure 4b). In addition, IFN-*γ* treatment strikingly increased phosphorylation of STAT1, and AG490 pretreatment decreased phosphorylation of STAT1 by 55%, as expected (Figure 4c). To further confirm whether STAT1 phosphorylation affected CX3CL1 expression, fludarabine, a selective STAT1 inhibitor, was used.^24^ As shown in Supplementary Figure 2E, while fludarabine exhibited no influence on the amount of STAT1 protein, it inhibited STAT1 phosphorylation in a dose-dependent manner. Uterine stromal cells, after exposure to fludarabine at 100 *μ*M for 2 h, showed a loss of 60% and 50% of CX3CL1 and pSTAT1 (phosphorylated STAT1), respectively (Figure 4d), suggesting a close correlation between STAT1 activation and the production of CX3CL1. Collectively, these data strongly suggested that IFN-*γ* upregulated CX3CL1 expression through a JAK2-STAT1 pathway. We then next explored whether and how IFN-*γ*-driven CX3CL1 regulated NK cell migration.

### CX3CL1 facilitated peripheral NK cell migration

**Figure 5:**
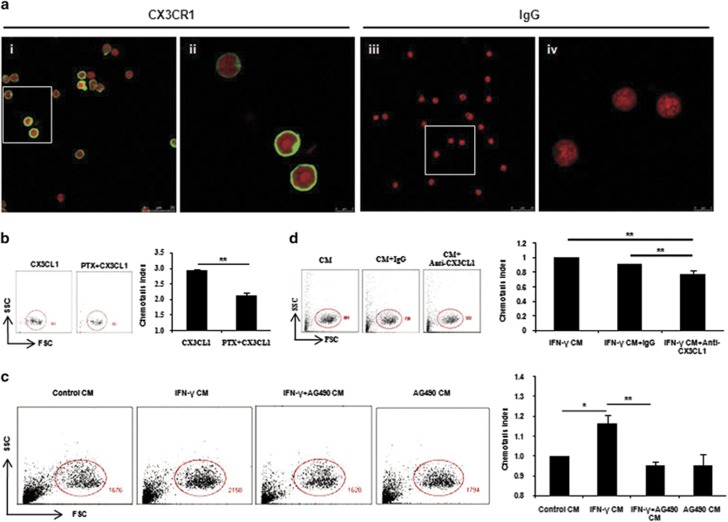
CX3CL1 facilitated peripheral NK cell migration. (**a**) Isolated splenic CD3^−^CD49b^+^ NK cells were spun onto slides, stained for CX3CR1 (green) or isotype IgG and counterstained with PI (red). Panels ii and vi show higher magnifications of the areas marked by the white rectangles in panels i and iii, respectively. Original magnification, × 63/1.40 (oil), zoom 1.00 (i and iii) and zoom 4.00 (ii and vi). Data shown are representative of two independent experiments from two mice. (**b**) To distinguish between chemotaxis and chemokinesis, NK cells were preincubated overnight with or without 500 ng/ml PTX before measuring cell migration. NK cells were gated on the basis of forward scatter and side scatter (FSC-SSC) and the numbers of NK cells were shown. Data show the mean±S.E.M. of three independent experiments. **P<0.01 by independent samples *T*-test. (**c**) After 12 h in culture, stromal cell CM (termed control CM), IFN-*γ*-treated stromal cell CM (termed IFN-*γ* CM), AG490-preincubated stromal cells before IFN-*γ* treatment CM (termed IFN-*γ*+AG490 CM) and AG490-treated stromal cell CM (termed AG490 CM) were collected and NK cell migration in response to them was measured as described above. For comparison, the chemotaxis index of NK cells toward control CM was set at 1. Data show the mean±S.E.M. of three independent experiments. *P<0.05 and **P<0.01 by one-way analysis of variance (ANOVA). (**d**) Five *μ*g/ml anti-CX3CL1-neutralizing mAb or control rat IgG was added to the NK cell suspension and the migration of NK cells toward IFN-*γ* CM was measured as described above. The chemotaxis index of NK cells without any treatment toward IFN-*γ* CM was set at 1. Data show the mean±S.E.M. of three independent experiments. **P<0.01 by one-way ANOVA

To explore whether CX3CL1 would induce the migration of NK cells, we first verified that peripheral NK cells expressed CX3CR1 at their surface. We performed immunostaining on NK cells, and observed a bright staining when cells were incubated with CX3CR1 Ab (Figure 5a). Then, the ability of peripheral NK cells to respond to CX3CL1 was assessed. Increasing the dose of CX3CL1 triggered a significant increase in cell migration; doses began at 100 ng/ml, and this effect reached a plateau at 500 ng/ml (Supplementary Figure 3A). Furthermore, an overnight preincubation of NK cells with pertussis toxin (PTX) at 500 ng/ml massively blocked the stimulatory effects of CX3CL1 (Figure 5b), indicating a CX3CL1-induced chemotaxis rather than chemokinesis.^25^

To mimic the uterine local environment, uterine stromal cells were isolated, cultured and used to prepare conditioned medium (CM). The chemotactic activity of the stromal cell CM (termed control CM) on NK cells was assessed. Chemotaxis to control CM increased robustly to 9.58-fold over that of the control group (Supplementary Figure 3B). Similarly, PTX partially abolished CM-mediated migration (data not shown). More importantly, we further found that chemotaxis to IFN-*γ*-treated stromal cell CM (termed IFN-*γ* CM) was significantly increased compared with control CM, and this enhancement of chemotaxis by IFN-*γ* CM can be significantly reversed by preincubation of stromal cells with AG490 (Figure 5c). To clarify the effect of CX3CL1 in IFN-*γ* CM, a neutralizing anti-CX3CL1 monoclonal antibody (mAb) was used. Blocking of CX3CL1 with neutralizing mAb could partially inhibit the migration of NK cells toward IFN-*γ* CM, compared with the addition of an isotypic IgG control (Figure 5d).

**Figure 6:**
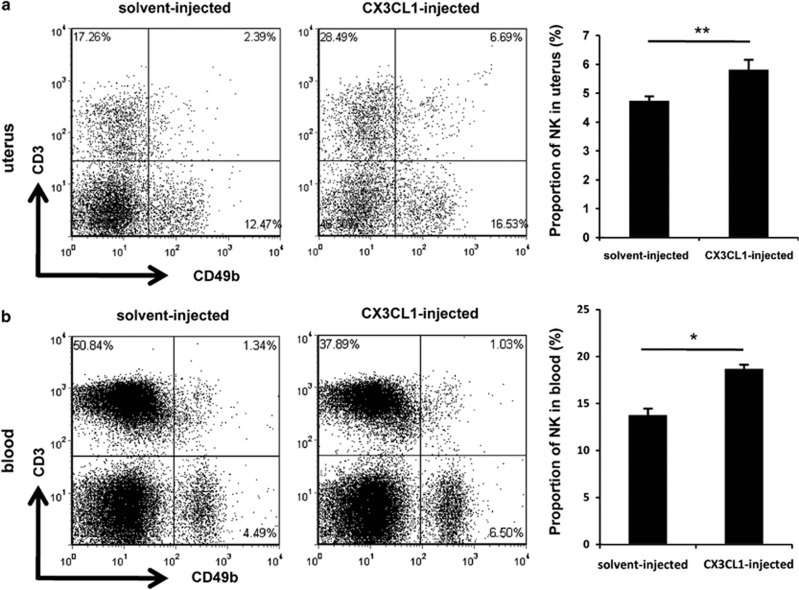
CX3CL1 facilitated CD49b^+^ NK cell migration *in vivo*. Homozygously mated BALB/c female mice were injected intraperitoneally with 1 *μ*g of CX3CL1 or placebo on GD6 and killed on GD8. Flow cytometric analysis of cell suspensions from the uteri (**a**) and peripheral blood (**b**). See Supplementary Figures 1B and C for the gating strategy and the percentages of CD3^−^CD49b^+^ NK cells (lower-right quadrant). Data show the mean±S.E.M. of three independent experiments and are obtained from three mice of each group, respectively. *P<0.05 and **P<0.01 by independent samples *T*-test

Although CX3CL1 induced robust migration of peripheral NK cells *in vitro*, this may not reflect the action of this drug *in vivo*. To verify the effect of CX3CL1 *in vivo*, recombinant mouse CX3CL1 was administered intraperitoneally to BALB/c females. Compared with mice treated with placebo, mice treated with CX3CL1 showed a higher percentage of CD3^−^CD49b^+^ NK cells in the uterus (Figure 6a). Similarly, a significant increase in CD3^−^CD49b^+^ NK cells was found in the peripheral blood of CX3CL1-treated mice (Figure 6b). Therefore, these data showed that CX3CL1 was associated with increased proportion of CD49b^+^ NK cells *in vivo*. Collectively, our above results strengthened the idea that upregulated uterine expression of CX3CL1 by IFN-*γ* was conducive for the NK cell uterine homing from the periphery.

## Discussion

IFN-*γ* has been widely evaluated as a potential mediator of pregnancy failure in humans.^1^ We describe here that IFN-*γ* can significantly increase uterine CX3CL1 expression via activation of the JAK2-STAT1 pathway, thus inducing CD49b^+^ NK cell uterine homing, and eventually provoke foetal loss in syngeneically mated BALB/c mice. To our knowledge, this is the first comprehensive study to correlate the deleterious effects of IFN-*γ* during pregnancy with the aberrant regulation of CX3CL1 and NK cells.

IFN-*γ* concentration per implantation site was prominent during early pregnancy in the mice, and uNK cells were the main source of IFN-*γ*.^10, 26^ As reported by Ashkar and Croy,^10^ IFN-*γ* concentration was ~4 U per implantation site on GD6 and peaked on GD10, with ~10 U per implantation site. Implantation sites of IFN-*γ*- and IFN-*γ*R*α*-null mice did not undergo normal gestation-induced spiral artery modification and contained elevated numbers of incompletely differentiated uNK cells and widespread necrotic deciduas, suggesting that IFN-*γ* contributed to the initiation of uterine vascular modifications, maturation of uNK cells and maintenance of decidual integrity.^27^ In this report, when each female mouse received an injection of 5000 U IFN-*γ* intraperitoneally on GD6, we observed a higher resorption rate on GD8. Thus, although IFN-*γ* had critical roles in successful pregnancy, a supraphysiological dose of IFN-*γ* was harmful to conceptus. However, the ovaries of IFN-*γ*-treated mice exhibited no overt histological abnormalities, suggesting that IFN-*γ* did not exert its effects on ovaries in this model.

The best studied mouse model of spontaneous foetal loss was the mating of CBA/J females with DBA/2 males.^3^ The abnormal resorption rate in the CBA/J × DBA/2 mating combination was thought to be because of activated NK cells and mononuclear cells expressing Mac-1 (CD11b) and F4/80.^7, 28^ Additionally, human RPL (recurrent pregnancy loss) is associated with NK cells.^29^ Our data indicated that the percentage of CD3^−^CD49b^+^ NK cells in the blood and uterus from the foetal resorption group was significantly increased. Thus, our results suggested that CD49b^+^ NK cells were incompatible with successful pregnancy, which seemed to be consistent with a previous report that the cytotoxicity of CD49b^+^ NK cells was higher than that of CD49b^−^ NK cells.^12^ Surprisingly, we observed no DBA-positive uNK cells in IFN-*γ*-induced abortion mice. Increasing evidence supports the idea that uNK cells provide major contributions to decidual and vascular remodelling.^27, 30^ Thus, necrotic deciduas and poor angiogenesis were found within the implantation sites of aborted mice that displayed the absence of uNK cells. At midgestation, excessively accumulated numbers of small, hypogranular uNK cells were found in IFN-*γ*^−/−^ or IFN-*γ*R*α*^−/−^ mice,^27^ leading us to hypothesize that increased apoptosis induced by IFN-*γ* may account for the absence of uNK cells in our model. Because there were also DBA^−^ uNK cells present,^31^ we could not exclude the possibility that DBA^−^ uNK cells may be present in IFN-*γ*-induced abortion mice.

NK cells, unlike T cells or B cells, which generate Ag-specific receptors by gene rearrangement, are the third major lymphocyte population.^32^ The earliest progenitors exclusively committed to NK cell lineage are characterized by the expression of CD122 in the mouse.^33^ After the acquisition of CD94-NKG2 receptors and Ly-49, NK cells acquire *α*_v_, CD49b and Mac-1 in order during maturation.^34^ Recently, it was reported that CD27 dissected mature NK cells into two subsets, with distinct responsiveness and migratory capacity in the mouse,^33^ suggesting that the different effects of IFN-*γ* on CD49b^+^ NK and DBA^+^ uNK cells may be because of their distinct responses to IFN-*γ*-driven CX3CL1. NK cells are regulated by activating and inhibitory cell surface receptors.^35^ By defining uNK cells as CD3^−^CD122^+^ cells, Yadi *et al.*^36^ reported two distinct subsets in the mice: a DBA-negative population was similar to peripheral NK cells, whereas a DBA-positive population had an unusual NKp46^+^NKG2D^+^NK1.1^−^CD49b^−^ phenotype, and a distinct Ly-49 receptor repertoire compared with CD49b^+^ NK cells.^36^ The two uNK subsets were different in functional potential with a biased gene expression.^37^ Other finding indicated that DBA^+^ and DBA^−^ uNK cells may represent cells arising from different sources, and DBA^+^ uNK cells may represent cells arising from homed progenitor or precursor cells.^31^ CD49b^+^ uNK cells were only a minor subset during normal pregnancy in the mouse,^36^ whereas IFN-*γ* administration increased the percentage of CD49b^+^ NK cells in our model. Therefore, these results raise the question whether IFN-*γ* alters the function and phenotype of CD49b^+^ NK cells. It was reported that transforming growth factor *β*1 favoured a transition from peripheral blood NK cells to uNK cells in humans.^38^ However, the relationships, in terms of differentiation between DBA^+^ and DBA^−^ uNK cells, still needed further experiments to elucidate in the mice.

Chemokines may have a fundamental role in forming a specialized immune milieu at the maternal–foetal interface by the recruitment of immune cells.^39, 40, 41^ Here, we provided multiple lines of evidences that CX3CL1 may contribute to CD49b^+^ NK cell recruitment to the uterus. First, we observed that upregulated uterine expression of CX3CL1 was associated with an increased proportion of CD49b^+^ NK cells in the uterus after IFN-*γ* administration *in vivo*. Moreover, IFN-*γ*-driven expression of CX3CL1 in uterine stromal cells was closely correlated with the increased migration of NK cells. In addition, the NK cell migration was significantly decreased when CX3CL1-neutralizing mAb was added to the CM, which clearly demonstrated that the chemotaxis of the uterine stromal cells CM was partially because of the presence of CX3CL1. Finally, we observed a greater increase in the recruitment of NK cells into the endometrium after exogenous CX3CL1 administration. Fraticelli *et al.*^20^ reported that functional CX3CR1 was expressed strongly in NK cells. In mice, CX3CR1 was identified at a late stage of NK cell development^18, 19^ and regulated NK cell activity *in vivo* via promoting NK cell trafficking.^42^ We verified that NK cells expressed CX3CR1 at their surface and, interestingly, that CX3CR1 expression was significantly upregulated in the uterus after IFN-*γ* treatment. The leucocytes may account for the increased CX3CR1 expression because we could not detect CX3CR1 in uterine stromal cells (data not shown). The unavailability of CX3CR1 Ab for flow cytometry restricted us from detecting CX3CR1 expression on NK cells directly *in vivo*. Nonetheless, our data seemed to support the idea that CD49b^+^ NK cells were recruited from peripheral NK cells, and a bias towards CD49b^+^ NK cells may lead to pregnancy failure. It was reported that CX3CR1- or CX3CL1-deficient mice did not exhibit any overt histological abnormalities and behavioural abnormalities.^43, 44^ It would be fascinating to investigate whether the responses of CX3CL1^−/−^ or CX3CR1^−/−^ mice to IFN-*γ* stimulus would be indistinguishable from those of wild-type mice.

In addition to CX3CL1,^17^ CCL3, CXCL10 and CXCL12 also regulated the trafficking of mouse NK cells.^45^ Our results showed that the expression of CXCL12, CXCL10, CCL4 and CCL5 was markedly stimulated by IFN-*γ* treatment in the uteri (data not shown). Compared with the addition of CX3CL1 alone, CM markedly increased the NK cell migration. Moreover, the addition of CX3CL1-neutralizing mAb to CM reduced NK cell migration by ~20%. Taken together, these results suggested that the uterus relied heavily on CX3CL1 to regulate the recruitment of NK cells and that other chemokines, such as CXCL10 and CXCL12, may also contribute to this phenomenon.

In summary, our data indicate that exogenous IFN-*γ* administration leads to the aberrant modulation of CD49b^+^ NK cells in the uterus via the upregulated expression of CX3CL1. We have now added chemokines, regulators of leucocyte trafficking,^15^ to the list of factors that cause IFN-*γ*-induced pregnancy failure. This may be a novel mechanism by which IFN-*γ* causes pregnancy failure and may provide a theoretic basis for human embryo abortion therapy.

## Materials and Methods

### Mice

Eight- to ten-week-old inbred BALB/c mice were purchased from Vital River Laboratories (VRL, Beijing, China). Mice, housed in a temperature- and humidity-controlled room with a constant photoperiod (12 L : 12 D), were fed *ad libitum* and had free access to tap water. Studies involving mouse usage were approved by the Institutional Animal Care and Use Committee of the Institute of Zoology, Chinese Academy of Sciences (Beijing, China). Pregnancy was achieved by caging female mice with a fertile male at a 2 : 1 ratio, and the day when a copulatory plug was observed was termed GD1.

### Treatment of mice with IFN-*γ* or CX3CL1 and histology

For IFN-*γ* treatment, homozygously mated BALB/c females were injected intraperitoneally with 5000 U IFN-*γ* (Peprotech, London, UK) or placebo (sodium phosphate containing 0.1% BSA) on GD6. For CX3CL1 treatment, homozygously mated BALB/c females were injected intraperitoneally with 1 *μ*g of CX3CL1 (R&D Systems, Minneapolis, MN, USA) or placebo (PBS containing 0.1% BSA) on GD6. Mice were killed by cervical dislocation on GD8, and foetal resorption was assessed by observing the contents of uterus. Mice without gross implantation sites or tissue debris in the uterus were considered not pregnant and excluded from the experiment.

For histological analysis, uteri and ovaries were removed and fixed in 4% paraformaldehyde (PFA) overnight at 4 °C. After fixation, tissues were treated with ethanol and xylene and embedded in paraffin. Sections of 5 *μ*m in thickness were prepared and stained using haematoxylin and eosin (H&E).

### Splenocyte transplantation

Spleens from placebo-treated or IFN-*γ*-induced abortion donor mice were gently homogenized in RPMI-1640 medium (HyClone, Logan, UT, USA) supplemented with 1% FBS (HyClone), penicillin/streptomycin (100 U/ml) and then sifted through a 37 *μ*m cell strainer. Donor cells were depleted of RBCs using an ammonium chloride lysing solution (0.14 M NH_4_Cl, 10 nM KHCO_3_ and 1 nM EDTA). Cells were then washed with RPMI-1640 medium and resuspended in RPMI-1640 medium. Homozygously mated BALB/c females on GD6 were used as recipients and were intravenously injected with 1 × 10^7^ donor cells in 100 *μ*l RPMI-1640. Two days later, recipients were killed, and the uteri were examined for the ratio of foetal abortions.

### Total RNA isolation and quantitative PCR

Total RNAs were extracted with a kit (BioTeke, Beijing, China) and then used as templates for reverse transcription (Promega, Madison, WI, USA). cDNA was amplified using SYBR Green MasterMix (ComWin Biotech Co. Ltd, Beijing, China) according to the manufacturer's instructions. Quantitative PCR was performed with a LightCycler 480 (Roche, Indianapolis, IN, USA). The primers used are summarized in Supplementary Tables 1 and 2, and the target gene mRNA expression was normalized to glyceraldehyde-3-phosphate dehydrogenase (GAPDH) expression. The fold change was calculated as 2 ^−ΔΔCt^ (cycle threshold).

### Western blotting

The following primary Abs were used: anti-CD49b, anti-STAT-1, anti-pSTAT-1 (Cell Signalling Technology Inc., Danvers, MA, USA), anti-CX3CL1 (eBioscience, San Diego, CA, USA), anti-CX3CR1 (eBioscience), anti-actin (Santa Cruz Biotechnology, Santa Cruz, CA, USA) and anti-GAPDH (Hangzhou Goodhere Biotechnology Co. Ltd, Hangzhou, China). Proteins were extracted by nondenaturing lysis buffer (Applygen, Beijing, China), and the concentration was determined by a bicinchoninic acid Protein Assay Kit (Pierce, Rockford, IL, USA). Proteins were separated by SDS–PAGE and transferred onto a nitrocellulose membrane (Pall, New York, NY, USA). The membranes were blocked in 5% skimmed dry milk in TBST at 37 °C for 1 h and then incubated with primary Abs at 4 °C overnight, followed by incubation with secondary Abs conjugated to HRP at 37 °C for 1 h (KPL, Gaithersburg, MD, USA). Chemiluminescence reactions were performed with an ECL Detection Kit (Pierce), and images were acquired using a Kodak X-Omat film (Carestream, Xiamen, China). Bands were analysed using Bio-Rad Quantity One software (Bio-Rad, Hercules, CA, USA), and expression was calculated as the ratio of the signal for the specific protein to the signal for actin or GAPDH.

### Immunochemical staining

Anti-CX3CL1 primary Ab and biotinylated-DBA lectin (Sigma-Aldrich, St. Louis, MO, USA) were used. Cryosections (8 *μ*m) of uteri were fixed in 4% PFA for 15 min. After being washed in PBS, the sections were blocked with 3% hydrogen peroxide for 5 min and sequential 10% horse normal serum (ZSGB-BIO, Beijing, China) at 37 °C for 1 h. Then, the cryosections were incubated with anti-CX3CL1 primary Ab or biotinylated-DBA lectin at 4 °C overnight, followed by incubation with secondary Ab conjugated to HRP or streptavidin-HRP (ZSGB-BIO) at 37 °C for 1 h.

The isolated uterine stromal cells were cultured for 12 h and fixed in 4% PFA for 15 min. After being washed in PBS, the cells were blocked with PBS containing 1% BSA at 37 °C for 1 h and then incubated with anti-CX3CL1 primary Ab at 4 °C overnight, followed by incubation with a secondary Ab conjugated to HRP at 37 °C for 1 h.

Cell and tissue slides were stained with diaminobenzidine (ZSGB-BIO) and counterstained with haematoxylin. Images were taken using a Nikon ECLIPSE Ni-U microscope and the NIS software (Nikon, Tokyo, Japan).

### Isolation and primary culture of uterine stromal cells

Uterine stromal cells were isolated from non-pregnant BALB/c mice, according to the protocol previously described, with minor modifications.^46^ In brief, uteri were dissected longitudinally and minced into small fragments. Uterine pieces were then placed in 1% trypsin (Sigma-Aldrich) and incubated in a sequence for 1 h at 4 °C and 1.5 h at room temperature with pipetting up and down every 10 min. The tissues remaining after the digestion were washed two times with PBS and incubated for subsequent digestion in 0.1% collagenase (Sigma-Aldrich) at 37 °C for 1 h with pipetting up and down every 10 min. At the end of the digestion, tissues were immediately diluted in DMEM/F12 at a 1 : 1 ratio (HyClone) with 10% FBS and mixed thoroughly. Then, the digested cells (primarily stromal cells) were sifted through 76 and 37 *μ*m cell strainers in a sequence and centrifuged. The pellet was washed two times with PBS. Cells were seeded at a density of 10^6^ cells per 35 cm^2^ in a dish containing DMEM/F12 (1 : 1) supplemented with 10% FBS. After uterine stromal cells adhered to the culture dishes, cells were transferred to serum-free DMEM/F12 (1 : 1) and starved for 24 h before treatment with various concentrations of IFN-*γ*. For inhibitor pretreatment, cells were incubated with various concentrations of AG490 (Sigma-Aldrich) or fludarabine (Selleck Chemicals, Houston, TX, USA) for 2 h before IFN-*γ* stimulation. After 12 h in culture, stromal cell CM was removed, centrifuged at 12 000 r.p.m. for 7 min and stored at −20 °C before use. The purity of isolated uterine stromal cells was above 90% (data not shown).

### Isolation of splenic NK cells

NK cells were aseptically isolated by mechanical dispersion of the whole GD8 BALB/c spleen in RPMI-1640 medium supplemented with 1% FBS and penicillin/streptomycin (100 U/ml). Cell suspensions were subsequently passed through a 37 *μ*m nylon mesh followed by density gradient separation using HISTOPAQUE 1083 (Sigma-Aldrich), according to the manufacturer's instructions. Briefly, cell suspensions were carefully loaded onto the HISTOPAQUE 1083 surface and centrifuged at 2000 r.p.m. for exactly 30 min at room temperature. After centrifugation, the opaque interface containing the mononuclear cells was carefully aspirated, washed with RPMI-1640 medium and resuspended in PBS containing 0.2% BSA. Cells were then pretreated with anti-mouse CD16/CD32 mAb (eBioscience) for 10 min on ice and incubated with APC-conjugated anti-CD49b (BD Biosciences, San Jose, CA, USA) and PE-conjugated anti-CD3 (eBioscience) mAbs for 30 min at 4 °C. After incubation, cells were washed once with PBS containing 0.2% BSA and sorted by a FACSAria instrument (BD Biosciences, Franklin Lakes, NJ, USA). Postsort NK cell purity was routinely more than 95% (data not shown).

### Chemotaxis assay

Quantitative NK cell transmigration assays were evaluated in 5 *μ*m pore Transwell inserts (Corning, Corning, NY, USA), as described previously.^39^ Splenic CD3^−^CD49b^+^ NK cells in 100 *μ*l were loaded in the upper well, and 600 *μ*l of medium supplemented with various concentrations of CX3CL1 or stromal cells CM was added to the lower compartment. Cells were allowed to migrate for 2.5 h at 37 °C, with 5% CO_2_, and then cells in the bottom chamber were collected and counted for 150 s using a FACScalibur (BD Biosciences, Franklin Lakes, NJ, USA). For treatment with PTX, cells were pretreated with PTX at 500 ng/ml overnight before the assay. Where indicted, blocking of CX3CL1 was performed by adding 5 *μ*g/ml anti-CX3CL1-neutralizing mAb (R&D Systems) or control rat IgG (R&D Systems) to the cell suspension. Cell migration was expressed as a chemotaxis index, which was calculated by the number of migrated cells in the presence of a given CX3CL1 concentration or stromal cell CM divided by the number of migrated cells in response to medium alone.

### Immunofluorescence

Isolated splenic CD3^−^CD49b^+^ NK cells were cytospun onto slides, air dried and fixed in 4% PFA for 20 min. Slides were then blocked with 10% horse normal serum at 37 °C for 1 h and incubated with rabbit anti-CX3CR1 or control rabbit IgG overnight at 4 °C. After three washes with PBS, slides were incubated with FITC-conjugated anti-rabbit secondary Ab (KPL) at 37 °C for 1 h. After staining, slides were washed with PBS and then mounted with antifade mounting media containing PI. Confocal microscopy was performed on a Leica TCS SP8 (Leica, Mannheim, Germany) and final image processing was performed using Leica Application Site Advanced Fluorescence (Leica).

### Cell suspension preparation and flow cytometry analysis

Peripheral blood was collected in heparinized disposable vacuum blood collection tubes. RBCs were lysed with an ammonium chloride lysing solution. Cells were then washed with RPMI-1640 medium and resuspended in PBS containing 0.2% BSA for further staining.

Uteri were dissected free from the mesometrium and minced into small fragments. Minced uteri were then placed in HBSS containing 200 U/ml hyaluronidase (Sigma-Aldrich), 1 mg/ml collagenase type IV (Sigma-Aldrich) and 0.2 mg/ml DNase (Sigma-Aldrich) for 20 min at 37 °C, as previously described with some modifications.^47^ After the digestion, cells were washed with PBS containing 0.2% BSA and incubated in the same buffer for 15 min at 37 °C before filtration through a 37 *μ*m nylon mesh. After centrifugation, cells were resuspended in PBS containing 0.2% BSA for further staining.

Cells suspensions were blocked with anti-mouse CD16/CD32 mAb and then incubated with APC-conjugated anti-CD49b plus FITC-conjugated anti-CD45 (eBioscience) and PE-conjugated anti-CD3 mAbs for 30 min at 4 °C. After staining, cells were rinsed with PBS containing 0.2% BSA and analysed on a FACScalibur.

### Statistics

Statistical significance was established when *P*<0.05. All statistical analyses were performed using SPSS version 16.0 (SPSS, Chicago, IL, USA).

## Abbreviations

Ab: antibody
CM: conditioned medium
Ct: cycle threshold
CX3CR1: CX3CL1 receptor
DAB: diaminobenzidine
DBA: *Dolichos biflorus agglutinin*
ECs: endothelial cells
GAPDH: glyceraldehyde-3-phosphate dehydrogenase
GD: gestational day
H&E: haematoxylin and eosin
IFN-*γ*: interferon gamma
JAK: Janus family kinase
KLRG1: killer lectin-like receptor G1
MHC: major histocompatibility complex
MLAp: mesometrial lymphoid aggregate of pregnancy
NK: natural killer
PFA: paraformaldehyde
pSTAT1: phosphorylated STAT1
PTX: pertussis toxin
RPL: recurrent pregnancy loss
STAT: signal transducers and activators of transcription
TGF*β*1: transforming growth factor *β*1
uNK: uterine NK
