# Supplementary material for: IFN-γ induces aberrant CD49b+ NK cell recruitment through regulating CX3CL1: a novel mechanism by which IFN-γ provokes pregnancy failure
Source: Cell Death Dis. 2014 Nov 6;5(11):e1512–. doi: 10.1038/cddis.2014.470 (PMC4260728; doi:10.1038/cddis.2014.470)
Supplement: Supplementary Information [file cddis2014470x1.doc]

**Supplementary Figures 1** (A) CD49b expression was analyzed by Quantitative PCR (*top panel*) and Western blotting (*bottom panel*) in uteri from solvent-injected and IFN-γ-injected mice on GD7. Data show mean ± SEM of four independent experiments and are obtained from four mice of each group respectively. *P<0.05, **P<0.01 by Independent-samples T test. (B) Dot plots shown are gating strategy to analyze NK cells in the uterus. Pan leucocytes are gated using anti-CD45 antibody versus SSC (ii) and then back gate analysis of NK cells (iii, iv) is shown. NK cells are defined as CD3^-^CD49b^+^. (C) Dot plots shown are gating strategy to analyze NK cells in the blood. Pan leucocytes are gated using anti-CD45 antibody versus SSC (ii). CD45 positive cells are then dissected based on CD3 and CD49b expression (iii). NK cells are defined as CD3^-^CD49b^+^.

**Supplementary Figures 2** (A) CX3CL1 expression was analyzed by immunofluorescence staining in uteri on GD8. Cryosections (8 μm) were incubated with antibody against CX3CL1 (green) and counterstained with PI (red). Original magnification ×40/1.10 (water) zoom 1.00. (B) CX3CL1 expression was analyzed by Western blotting in uteri from solvent-injected and IFN-γ-injected mice on GD7. Data show mean ± SEM of four independent experiments and are obtained from four mice of each group. *P<0.05 by Independent-samples T test. (C) Uterine stromal cells were treated with IFN-γ at doses of 10, 100, 250, 500 U/mL for 12 h, and then CX3CL1 protein expression was analyzed by Western blotting. Data show mean ± SEM of three independent experiments. *P<0.05 by One-way ANOVA. (D) Uterine stromal cells were treated with IFN-γ at a dose of 250 U/mL, and then CX3CL1 mRNA expression was analyzed by Quantitative PCR (*top panel*) and RT-PCR (*bottom panel*) at various time points. Data show mean ± SEM of three independent experiments. *P<0.05 by One-way ANOVA. (E) Uterine stromal cells were pretreated with fludarabine at 25, 50, 100 μM for 2 h before IFN-γ treatment, and then pSTAT1 and STAT1 were analyzed by Western blotting. STAT1, pSTAT1 were normalized to GAPDH, STAT1 respectively. Data show mean ± SEM of three independent experiments. *P<0.05, **P<0.01 by One-way ANOVA. GE, glandular epithelium; LE, luminal epithelium.

**Supplementary Figures 3** (A) Primary splenic NK cells migration in response to increasing doses of CX3CL1 was measured. NK cells were gated on the basis of FSC-SSC and the numbers of NK cells were showed. Data show mean ± SEM of three independent experiments. **P<0.01 by One-way ANOVA. (B) After 12 h in culture, stromal cells CM (termed Control CM) was collected, and then NK cells migration in response to Control CM was measured. Serum-free DMEM/F12 1:1 was used as Control. NK cells were gated on the basis of FSC-SSC and the numbers of NK cells were showed. Data show mean ± SEM of five independent experiments. **P<0.01 by Independent-samples T test.
