# Supplementary figures and images for: IFN-γ induces aberrant CD49b+ NK cell recruitment through regulating CX3CL1: a novel mechanism by which IFN-γ provokes pregnancy failure
Source: Cell Death Dis. 2014 Nov 6;5(11):e1512–. doi: 10.1038/cddis.2014.470 (PMC4260728; doi:10.1038/cddis.2014.470)

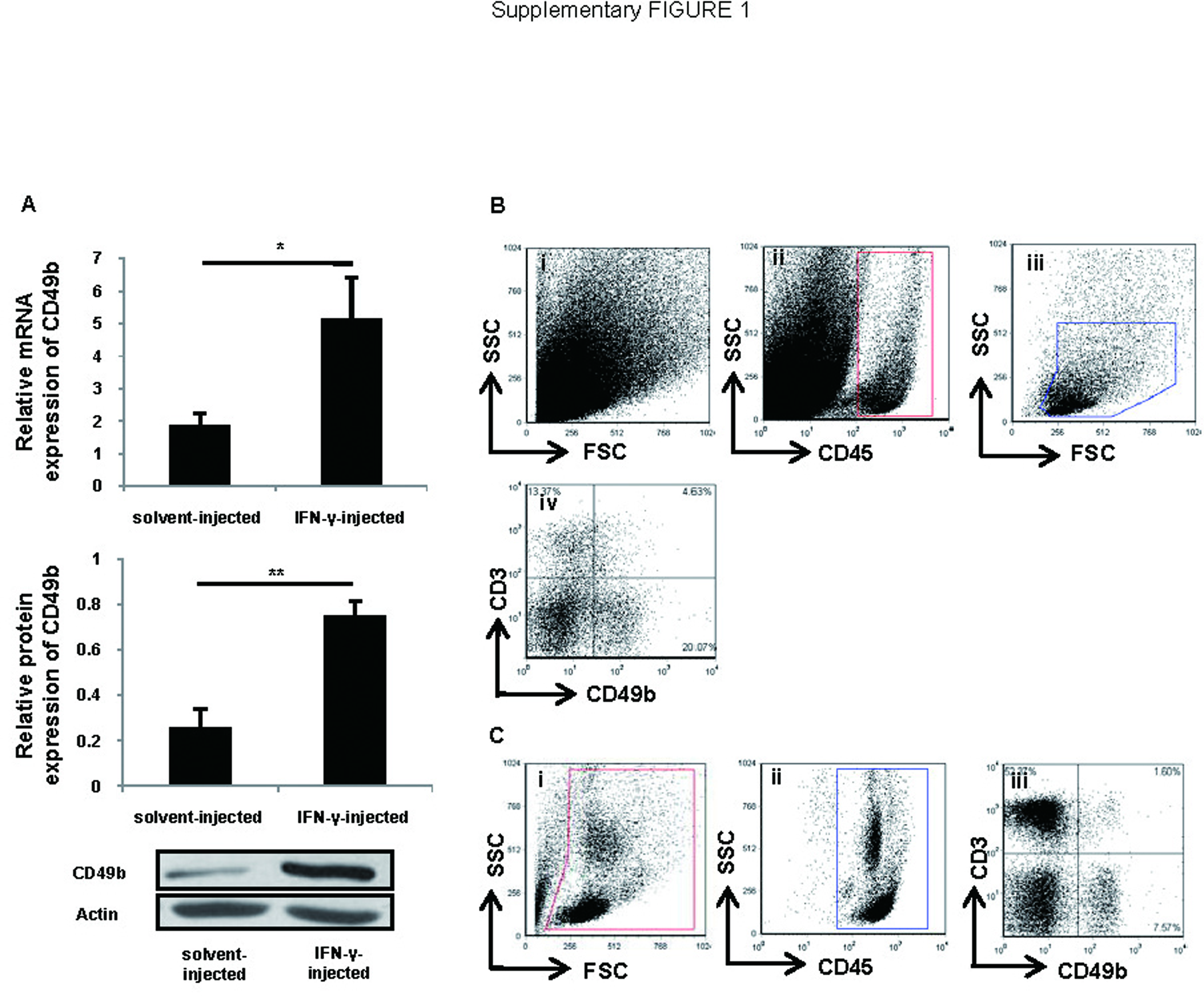

Supplement: Supplementary Figure 1 [file cddis2014470x2.tif]

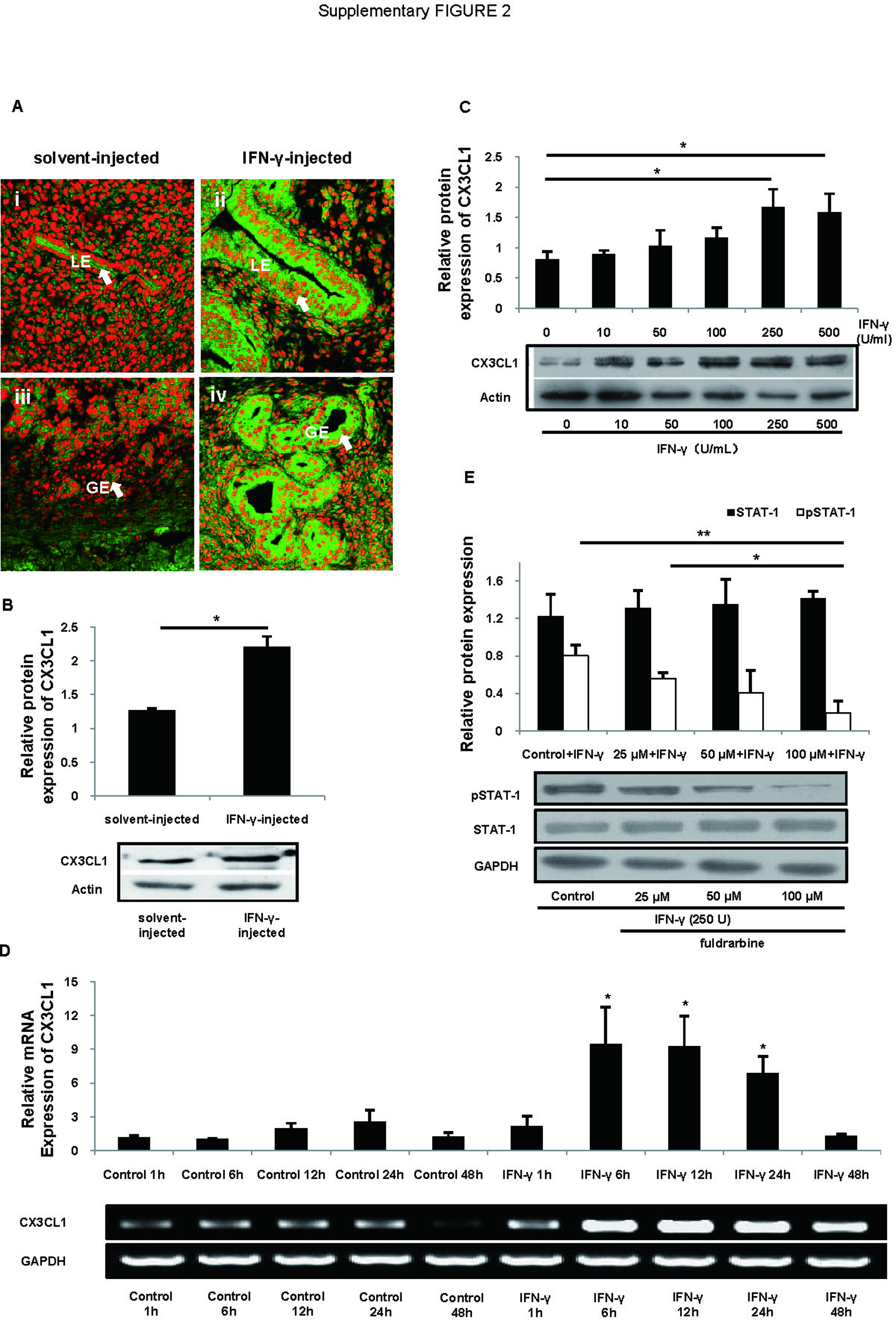

Supplement: Supplementary Figure 2 [file cddis2014470x3.tif]

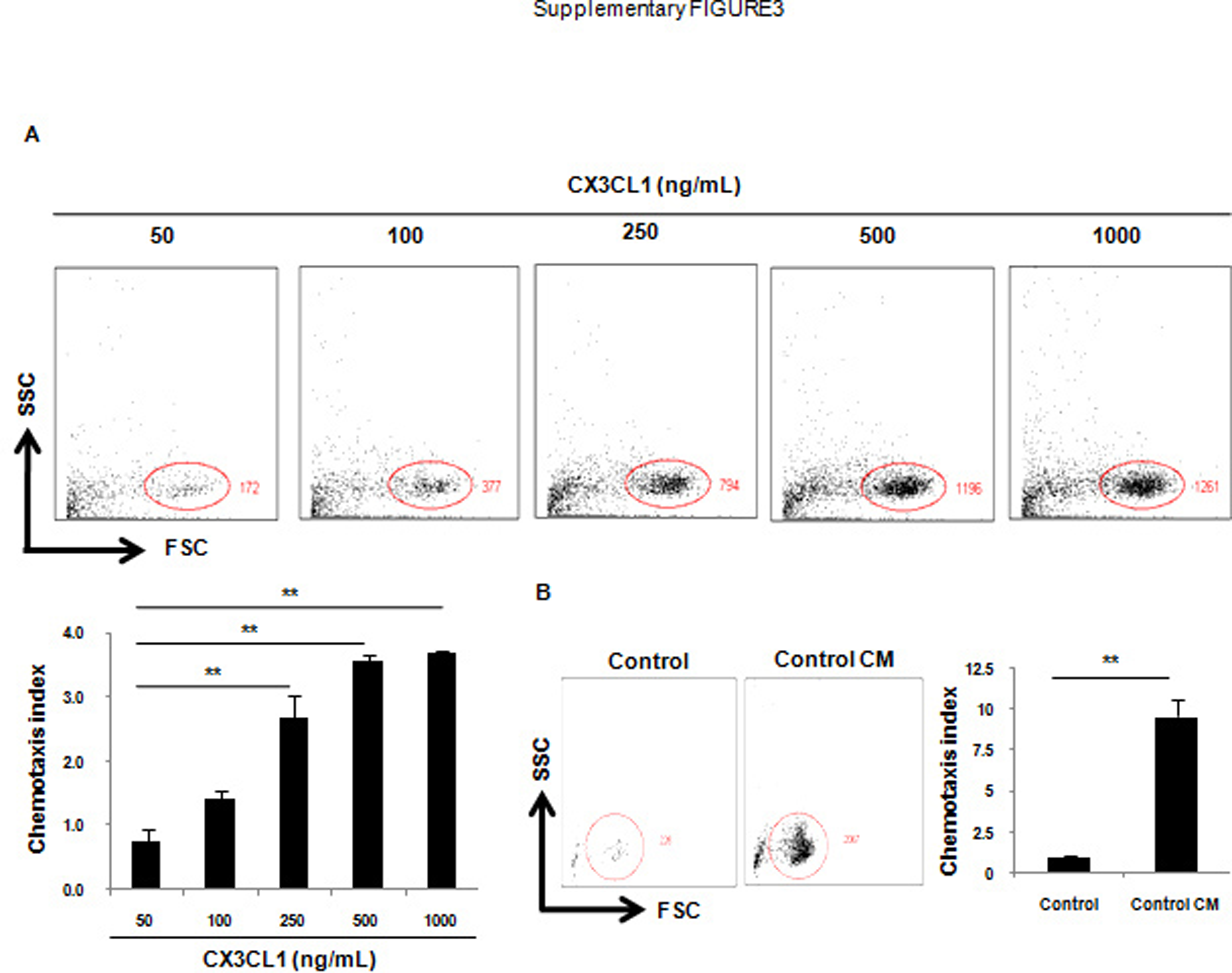

Supplement: Supplementary Figure 3 [file cddis2014470x4.tif]

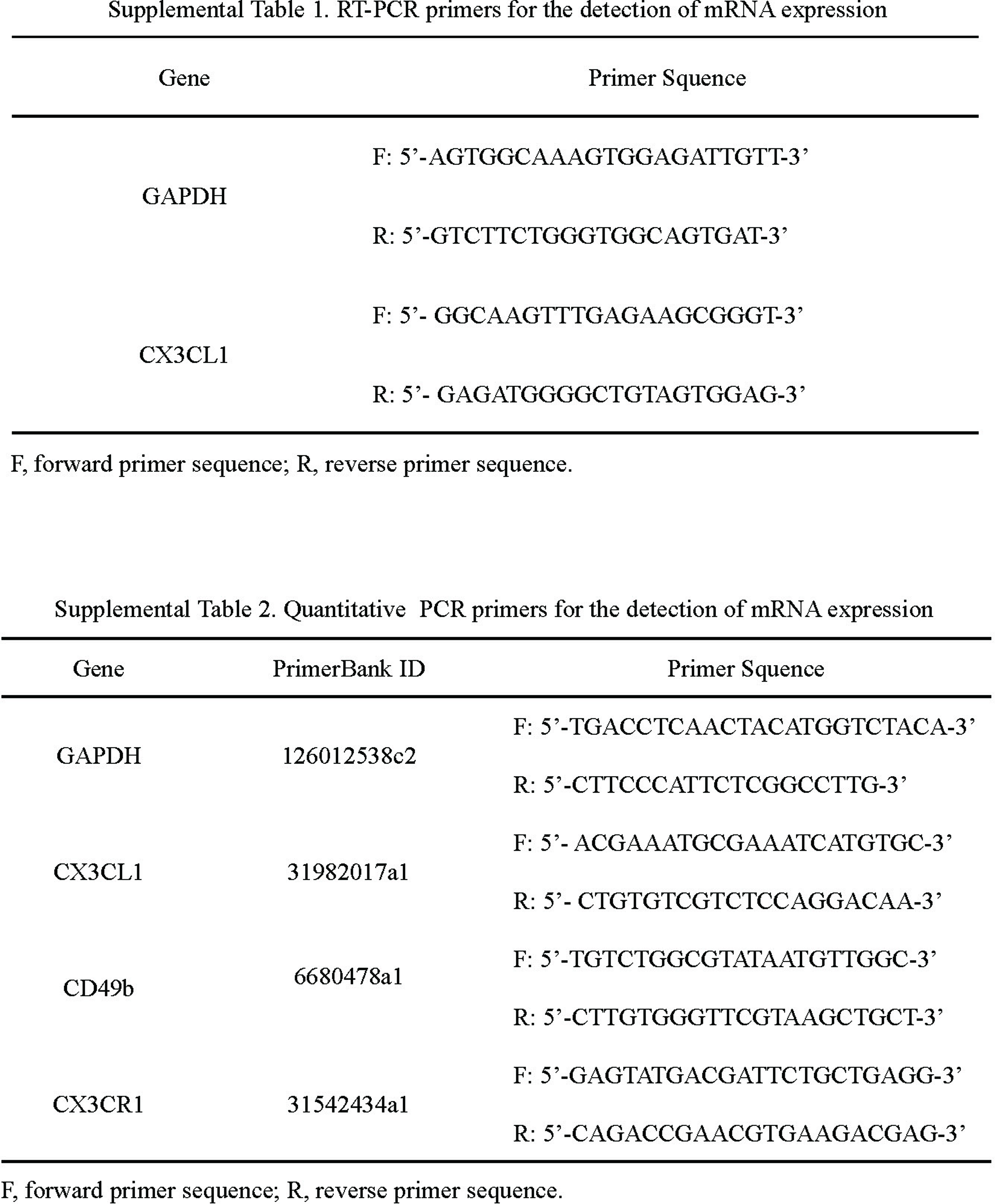

Supplement: Supplementary Table 1 [file cddis2014470x5.tif]
